# Influence of Heat Treatment on Precipitate and Microstructure of 38CrMoAl Steel

**DOI:** 10.3390/ma18153703

**Published:** 2025-08-06

**Authors:** Guofang Xu, Shiheng Liang, Bo Chen, Jiangtao Chen, Yabing Zhang, Xiaotan Zuo, Zihan Li, Bo Song, Wei Liu

**Affiliations:** 1 School of Metallurgical and Ecological Engineering, University of Science and Technology Beijing, Beijing 100083, China; 2 Wuhu Xinxing Ductile Pipe Co., Ltd., Wuhu 241002, China; 3 HBIS Material Technology Research Institute, Shijiazhuang 052160, China

**Keywords:** precipitate, microstructure, hot ductility, 38CrMoAl steel

## Abstract

To address the central cracking problem in continuous casting slabs of 38CrMoAl steel, high-temperature tensile tests were performed using a Gleeble-3800 thermal simulator to characterize the hot ductility of the steel within the temperature range of 600–1200 °C. The phase transformation behavior was computationally analyzed via the Thermo-Calc software, while the microstructure, fracture morphology, and precipitate characteristics were systematically investigated using a metallographic microscope (MM), a field-emission scanning electron microscope (FE-SEM), and transmission electron microscopy (TEM). Additionally, the effects of different holding times and cooling rates on the microstructure and precipitates of 38CrMoAl steel were also studied. The results show that the third brittle temperature region of 38CrMoAl steel is 645–1009 °C, and the fracture mechanisms can be classified into three types: (I) in the α single-phase region, the thickness of intergranular proeutectoid ferrite increases with rising temperature, leading to reduced hot ductility; (II) in the γ single-phase region, the average size of precipitates increases while the number density decreases with increasing temperature, thereby improving hot ductility; and (III) in the α + γ two-phase region, the precipitation of proeutectoid ferrite promotes crack propagation and the dense distribution of precipitates at grain boundaries causes stress concentration, further deteriorating hot ductility. Heat treatment experiments indicate that the microstructures of the specimen transformed under water cooling, air cooling, and furnace cooling conditions as follows: martensite + proeutectoid ferrite → bainite + ferrite → ferrite. The average size of precipitates first decreased, then increased, and finally decreased again with increasing holding time, while the number density exhibited the opposite trend. Therefore, when the holding time was the same, reducing the cooling rate could increase the average size of the precipitates and decrease their number density, thereby improving the hot ductility of 38CrMoAl steel.

## 1. Introduction

According to Chinese standards (GB/T 3077-2015) [1], 38CrMoAl steel is an alloy structural steel with an aluminum content as high as 1 wt%, which is mainly used for gears [2], hydraulic plungers [3], and carrier aircraft [4]. This steel tends to cause nozzle clogging during continuous casting due to its high Al content. Meanwhile, casting billets are prone to metallurgical defects such as cracks and component segregation [5]. The industrial production route for Φ600 mm 38CrMoAl steel at a domestic factory is as follows: Linz–Donawitz (LD) → ladle furnace (LF) → RH vacuum degassing (RH) → continuous cast (CC) → rapid forging → diameter forging. However, the cooled continuous casting billets in production have central cracks, which seriously affect their quality and subsequent processing.

The initiation and propagation of cracks in continuous casting billets are intimately linked to the high-temperature mechanical properties of steel. Generally, cracks are related to the hot ductility of steel [6,7,8,9,10,11,12,13], which influences the steel’s fatigue properties [14] and internal quality [15,16]. For high-Al steel grades, the existing research on cracking mechanisms has primarily focused on non-metallic inclusions [17,18], the strain rate and cooling rate [10,17,19], micro-alloying elements [9,19], and harmful elements (S, N, and P) [8,19,20]. In other steel types, such as API X60 micro-alloyed steel, the hot ductility is related to the austenite grain size and the size and volume fraction of precipitated phases [21]. For alloy structural steels such as 2.25Cr-1Mo, the hot ductility is associated with precipitated phases and ferrite [22,23,24]. Meanwhile, a number of advanced hot ductility models also offer valuable research orientations for crack prediction [25,26]. The hot ductility of steel reveals its deformation behavior and fracture mechanism at high temperatures, providing a theoretical basis for predicting cracks in continuous casting billets, reducing processing defects, and optimizing processes.

However, systematic data about the microstructure and precipitates near the fracture on the hot ductility of industrial-grade 38CrMoAl continuous casting billets remain scarce. To address this gap and improve the cracks, this study investigated 38CrMoAl steels from continuous casting billets using a Gleeble-3800 testing machine to characterize their hot ductility across the temperature range of 600–1200 °C. The aim was to guide the industrial production of 38CrMoAl continuous casting billets with high yield. Meanwhile, the effects of different holding times and cooling rates on the precipitates and microstructure of 38CrMoAl steel were investigated.

## 2. Experimental Materials and Methods

### 2.1. Experimental Materials

**Figure 1:**
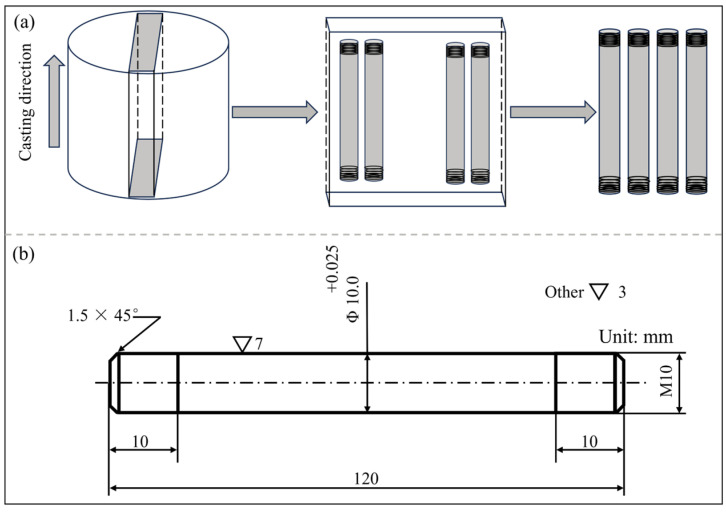
Diagram of the sample: (**a**) diagrams of sampling schematic; (**b**) tensile specimen.

Experimental samples were extracted from on-site continuous casting billets. Chemical analysis was performed via a Thermo Fisher ARL600 direct reading spectrometer (Waltham, MA, USA), and the total contents of oxygen and nitrogen were determined via a combined nitrogen and oxygen analyzer. The main chemical composition of the 38CrMoAl steel continuous cast billets is shown in Table 1 and a schematic diagram of the sample is shown in Figure 1. Specimens were sampled parallel to the casting direction, taken from the columnar crystal region of the billet at a position 10 mm below the billet surface to prevent interference from center segregation or shrinkage porosity on the test results. After processing, the tensile specimens had a dimension of Φ10 mm × 120 mm, with M10 threads machined at both ends. The surface roughness grade of the specimen’s working section was 7, while that of the remaining parts was 3.

### 2.2. Experimental Methods

(1) High-temperature Tensile Test

**Figure 2:**
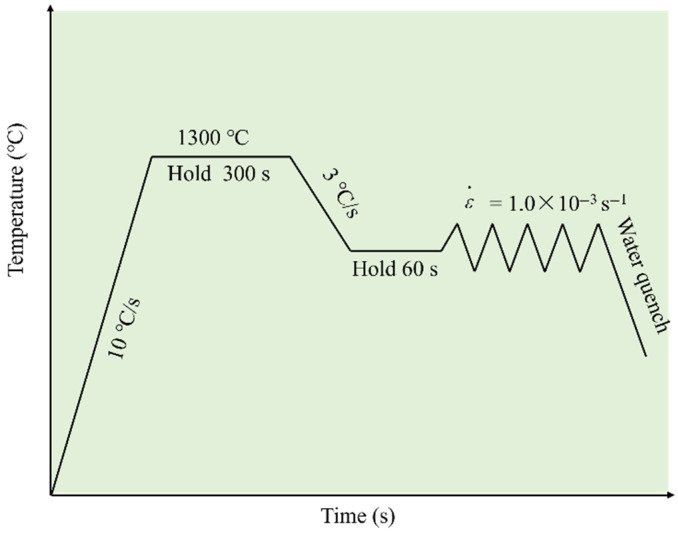
Thermal simulation test schedule of hot tensile tests.

The thermal simulation test schedule of hot tensile tests is shown in Figure 2. Specimens were heated to 1300 °C at a rate of 10 °C/s for 300 s, followed by controlled cooling at 3 °C/s to the target temperature range of 600–1200 °C. After a 60 s isothermal hold at each test temperature, tensile testing with the Gleeble-3800 (DSI, Segundo, CA, USA) was performed at a constant strain rate of 10^−3^ s^−1^ [17,23], and each temperature was tested in 3 times. Upon fracture, the specimens were immediately water-quenched to retain their high-temperature fracture morphology and microstructural features [27].

The reductions in area (RA) and tensile strength (*σ_b_*) were calculated using Formulas (1) and (2):

(1) $$RA=\frac{D_{0}^{2}-D_{1}^{2}}{D_{0}^{2}}\times 100\%$$

(2) $$\sigma_{b}=\frac{4F_{\max}}{{\pi D}_{0}^{2}}$$

where *D*_0_ is the original diameter of the sample, i.e., 10 mm; *D*_1_ is the diameter of the sample after fracture (mm); *F*_max_ is the maximum tensile force endured by the sample during the tensile process (N); and *σ_b_* is the tensile strength of the sample (MPa).

(2) Heat Treatment Experiment

**Figure 3:**
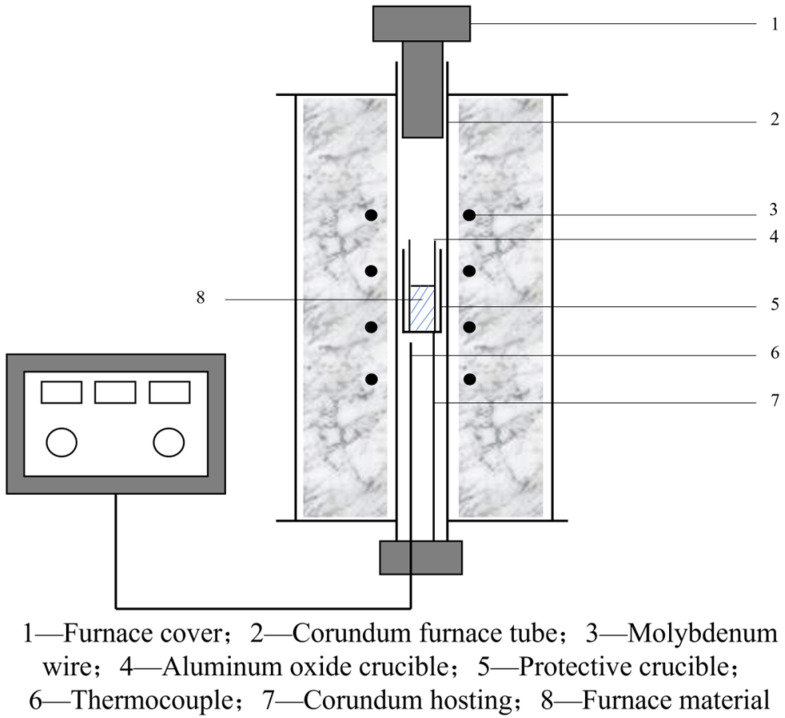
Structural diagram of high-temperature molybdenum wire furnace.

The high-temperature molybdenum wire furnace was heated to 950 °C. Metallographic specimens (10 mm × 10 mm × 10 mm) were placed in an alumina crucible and subjected to isothermal holding for 20 min, 40 min, 60 min, 80 min, and 100 min, respectively. After each holding period, partial specimens were subjected to water cooling and air cooling, respectively. Meanwhile, the remaining specimens were furnace cooled. Subsequently, the specimens were subjected to precipitate and microstructural analysis. The structural schematic of the high-temperature molybdenum wire furnace is presented in Figure 3.

(3) Characterization of Fracture Morphology, Microstructure, Grain Size, and Precipitated Phases

**Figure 4:**
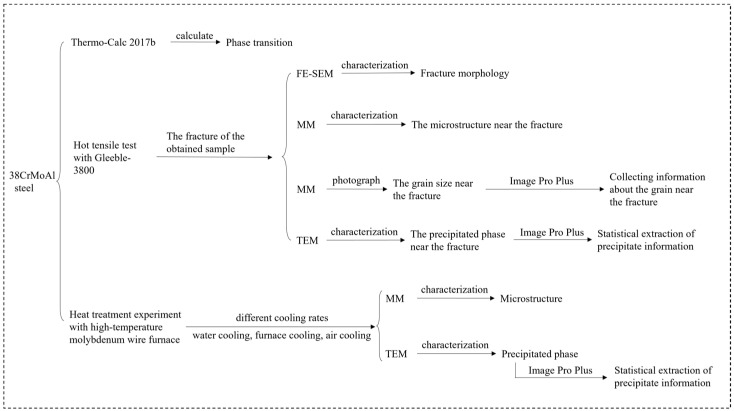
The equipment and characterization methods used in the experiment.

The fracture morphology was observed under a field-emission scanning electron microscope (FE-SEM, GeminiSEM300, ZEISS, Jena, Germany). Microstructural observations of both the fracture and heat-treated samples were conducted via a metallographic microscope (MM) after the specimens were subjected to pregrounding, polishing, and etching with a 4 vol% acid-alcohol solution. The grain sizes near the fracture were measured using the Image-Pro Plus software (Version 6.0.0.260). The Thermo-Calc 2017b software, combined with the TCFE v8.1 database and property diagram templet, was employed to simulate phase transformations in 38CrMoAl steel. Transmission electron microscopy (TEM, JEOL JEM-2010, Tokyo, Japan) operated at 200 kV and coupled with energy-dispersive spectroscopy (EDS), was used to characterize the morphology, size of precipitates, and chemical composition. The equipment and characterization methods used in the experiment are shown in Figure 4.

## 3. Results and Discussion

### 3.1. Hot Ductility

RA was used to measure the sample’s hot ductility [7,13,23,28,29], calculated using Formula (1). In this study, RA = 40% was taken as the critical threshold of the crack-sensitive zone during the continuous casting and straightening process [18,29,30]. When RA < 40%, it indicated that the steel grade was prone to cracking during continuous casting, and the corresponding temperature range was regarded as the brittle zone.

**Figure 5:**
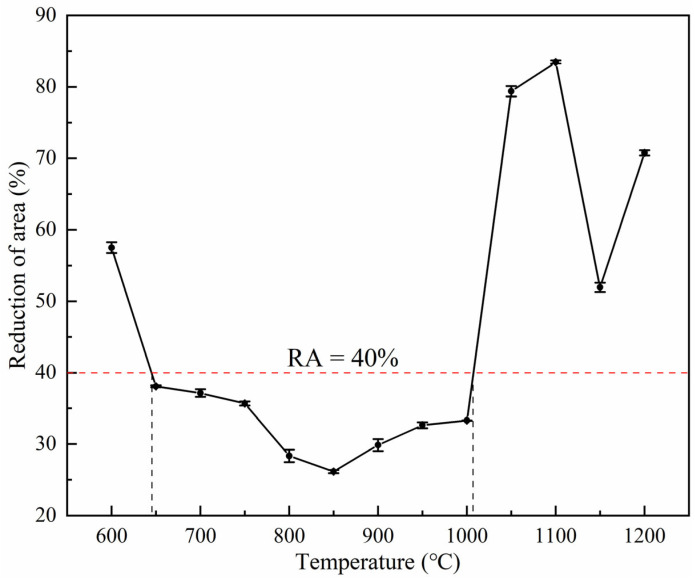
Reduction in area of 38CrMoAl steel specimens.

Figure 5 shows the hot ductility curve across varying temperatures. Within the test temperature range, no high-temperature brittle zone or the first brittle zone was detected. Given that the applied strain rate (1.0 × 10^−3^ s^−1^) was lower than the threshold (1.0 × 10^−2^ s^−1^) for the second brittle temperature region’s formation [13], this experimental condition excluded the occurrence of the second brittle zone. Based on the critical threshold of RA = 40% for the crack-sensitive region during continuous casting and straightening, the variation in the RA value with temperature can be divided into three categories: (I) In the low-temperature ductility region (600 °C), the RA value at 600 °C was approximately 57.5%, indicating favorable ductility at lower temperatures. (II) In the medium-temperature brittle region (645–1009 °C), the ductility trough spanned 645–1009 °C, with corresponding RA values ≤ 40%. The temperature range was identified as the primary brittle region where cracking susceptibility was highest during continuous casting. (III) In the high-temperature ductility region (1050–1200 °C), the RA value exhibited a non-monotonic trend, increasing first, then decreasing, and increasing again with rising temperature. All RA values exceeded 40%, with a peak of ~83.5% observed at 1100 °C.

### 3.2. Tensile Strength

**Figure 6:**
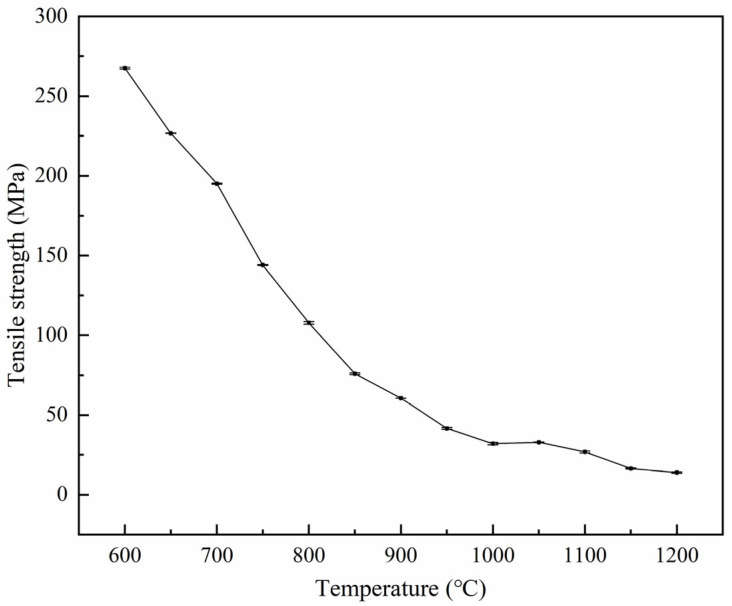
Tensile strength of 38CrMoAl steel specimens.

Figure 6 demonstrates that the tensile strength of 38CrMoAl steel exhibited a rapid decline in the 600–950 °C temperature range, followed by a slower decrease from 950 to 1200 °C. Notably, the peak tensile strength of ~267.5 MPa occurred at 600 °C, while the minimum value of ~13.9 MPa was recorded at 1200 °C.

### 3.3. Fracture Morphology

**Figure 7:**
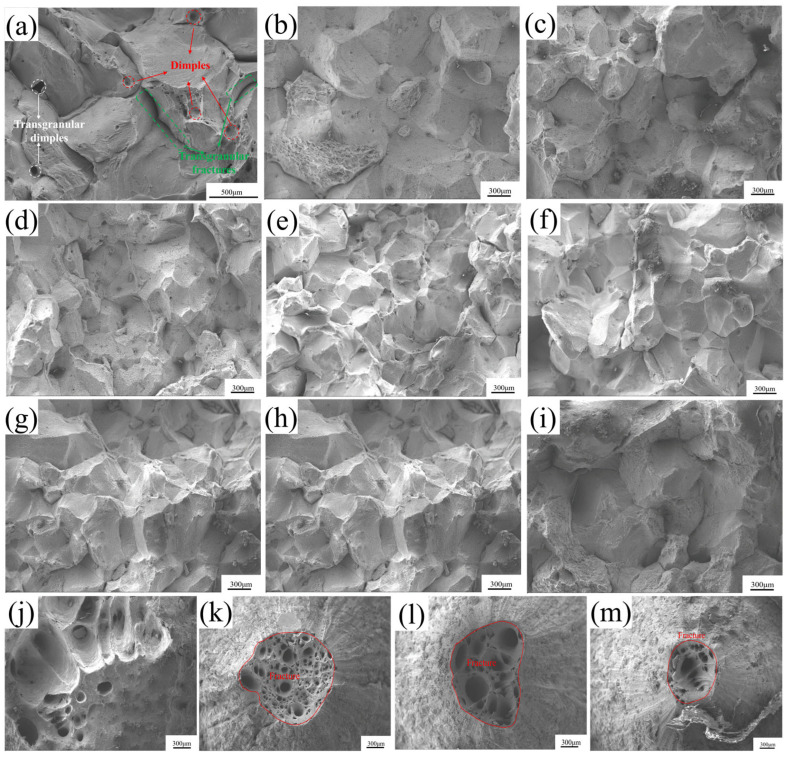
Fracture morphologies of the specimen after hot tensile testing at different temperatures: (**a**) 600 °C; (**b**) 650 °C; (**c**) 700 °C; (**d**) 750 °C; (**e**) 800 °C; (**f**) 850 °C; (**g**) 900 °C; (**h**) 950 °C; (**i**) 1000 °C; (**j**) 1050 °C; (**k**) 1100 °C; (**l**) 1050 °C; and (**m**) 1200 °C.

Figure 7 illustrates the fracture morphologies of specimens tested at 600–1200 °C. At 600 °C (Figure 7a), the fracture surface exhibited numerous pits of varying sizes and depths, characteristic of typical dimples that are particularly prominent at grain boundaries. This morphology indicated intragranular void formation during deformation, where voids grew and coalesced with increasing strain, leading to necking and eventual transgranular ductile fracture. As depicted in Figure 7b–d, within the temperature range of 650–1000 °C, the characteristics of brittle fracture on the fracture surface become more pronounced, exhibiting a granular morphology akin to sugar grains. Additionally, a limited number of dimples were observed on the fracture surface. Consequently, the fracture mode was identified as a combination of ductile fracture and intergranular brittle fracture. As illustrated in Figure 7e–h, the fracture surface of the sample exhibited a sugar-like appearance, characteristic of typical brittle fracture. As shown Figure 7i, the majority of the fracture surface displayed a sugar-like appearance, indicative of intergranular brittle fracture. Additionally, a limited number of dendritic structures, resulting from localized melting, were observed on the fracture surface. As shown in Figure 7j, within the temperature range of 1050–1200 °C, the fracture surface lost some morphological features due to excessive melting but still retained some large and deep dimples, indicating that ductile fracture had occurred. According to Figure 7k–m, the fracture surface showed large and deep dimples, indicating that ductile fracture had occurred.

### 3.4. Microstructural Characterization

**Figure 8:**
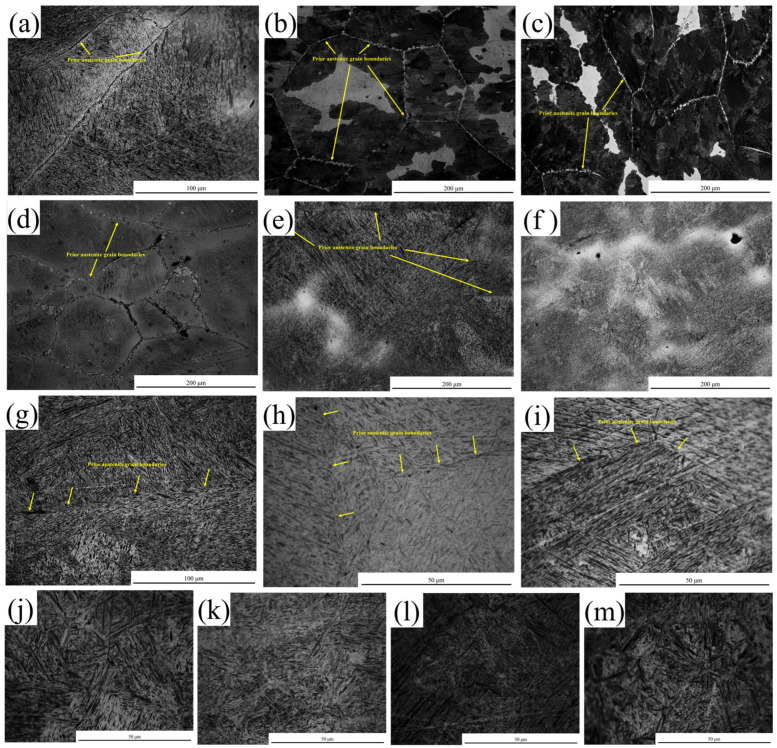
Microstructure near tensile fracture of the steel at different temperatures: (**a**) 600 °C; (**b**) 650 °C; (**c**) 700 °C; (**d**) 750 °C; (**e**) 800 °C; (**f**) 850 °C; (**g**) 900 °C; (**h**) 950 °C; (**i**) 1000 °C; (**j**) 1050 °C; (**k**) 1100 °C; (**l**) 1050 °C; and (**m**) 1200 °C.

Figure 8 shows the microstructure near the tensile fracture of the steel within the test temperature range of 600–1200 °C. At 600 °C, the microstructure had a Widmanstätten pattern. Meanwhile, continuous proeutectoid ferrite precipitated at the grain boundaries and distributed in a network pattern with uneven thickness, with an average thickness of approximately 2 μm, as shown in Figure 8a. At 650 °C and 700 °C, the microstructure was composed of a continuous network of proeutectoid ferrite precipitated along the grain boundaries, pearlite, and intragranular ferrite. The thickness of the proeutectoid ferrite was inconsistent, with average thicknesses of 2.2 μm and 3 μm, respectively, as shown in Figure 8b,c. At 750 °C, the microstructure was composed of a continuous network of proeutectoid ferrite precipitated along the grain boundaries, martensite, and a small amount of intragranular ferrite, and the average thickness of the proeutectoid ferrite was 3.8 μm, as shown in Figure 8d. At 800 °C, the microstructure consisted of the precipitation of proeutectoid ferrite and martensite, and the average thickness of the proeutectoid ferrite was 12 μm, as shown in Figure 8e. At 850 °C, the structure was composed of the precipitation of proeutectoid ferrite and martensite, and the average thickness of the proeutectoid ferrite was 15 μm, as shown in Figure 8f. Within the temperature range of 600–850 °C, the proeutectoid ferrite was distributed in a network pattern along the grain boundaries, which deteriorated the hot ductility of this steel. Meanwhile, the hot ductility of the specimen decreased with the increase in the average thickness of the proeutectoid ferrite. At 900–1200 °C, the microstructure of the specimen was austenite, which transformed into martensite after rapid cooling, as shown in Figure 8g–m.

### 3.5. Mechanism of Hot Ductility Evolution

Based on the RA values, temperatures of 600 °C, 850 °C, 950 °C, and 1100 °C were selected to investigate the effects of the microstructures and precipitated phases on hot ductility.

(i) Precipitate

**Figure 9:**
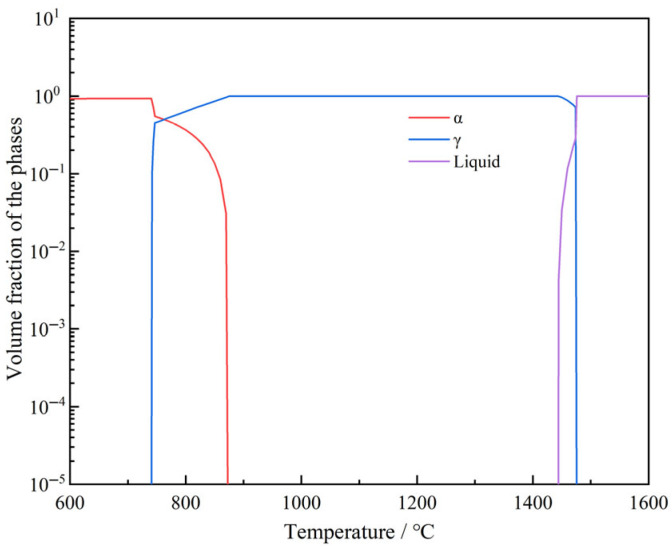
Equilibrium phase behavior in tested steel.

The characteristics of the phases of the experimental steel (the chemical composition of which is presented in Table 1) were calculated using Thermo-Calc, as shown in Figure 9. The precipitation characteristics of the liquid phase, austenite (γ), and ferrite (α) in the experimental steel within the temperature range of 400–1600 °C are listed in Table 2. As the temperature decreased from 1600 °C to 400 °C, the γ preferentially precipitated from the liquid phase at 1474 °C. When the temperature dropped to 870 °C, the γ began to transform into α. At 740 °C, the γ was completely transformed into α and disappeared entirely.

**Figure 10:**
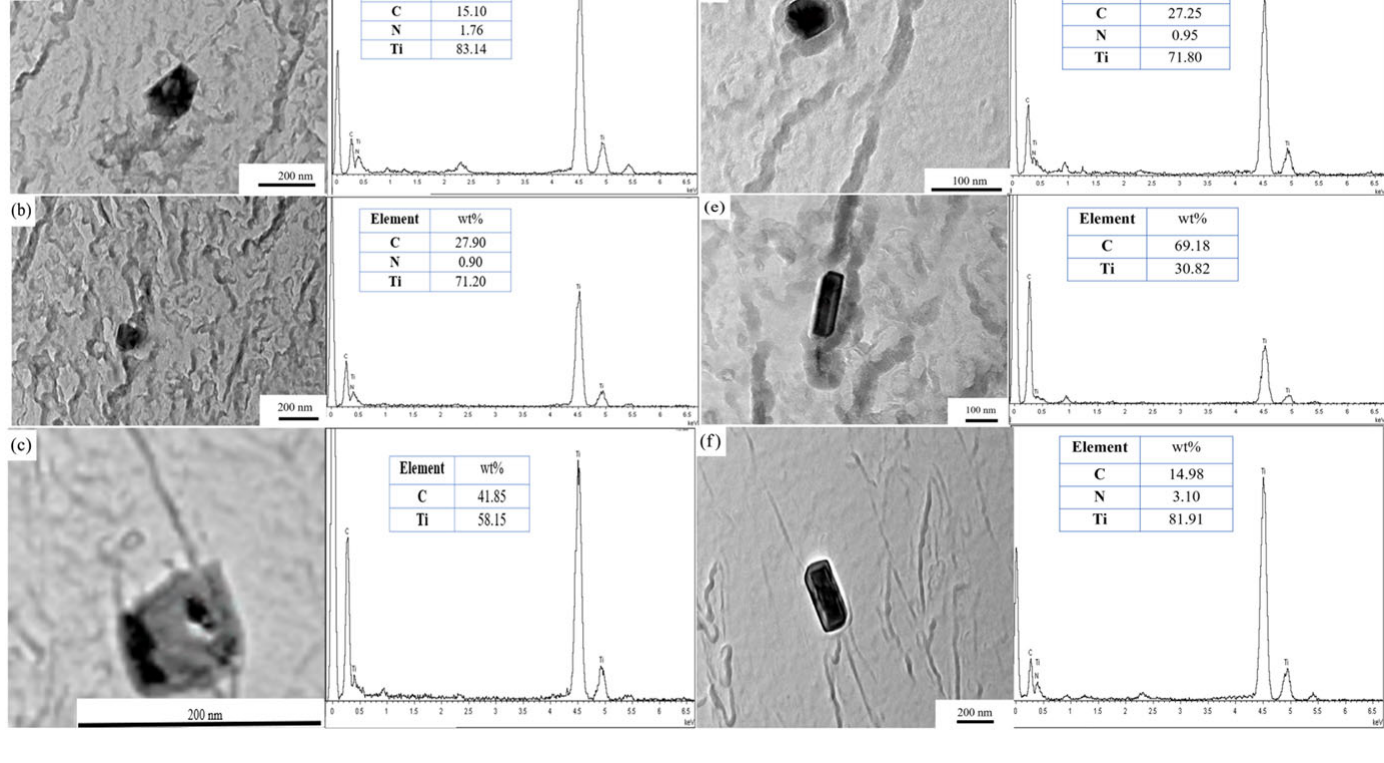
Morphology and composition of precipitates in the tested steels: (**a**) and (**b**) 600 °C; (**c**) 850 °C; (**d**) and (**e**) 950 °C; (**f**) 1100 °C.

Thermodynamic calculations showed that the initial precipitation temperatures of TiC, Ti(C,N), and AlN were 1444 °C, 1380 °C, and 1320 °C, respectively. As the nitrogen content in 38CrMoAl steel is extremely low (~0.0031 wt%), titanium (~0.0127 wt%) preferentially combines with nitrogen at high temperatures [19]. At elevated temperatures, TiC is preferentially formed due to its higher initial precipitation temperature. As temperature decreases, variations in the system’s free energy facilitate the repartitioning of carbon and nitrogen atoms: at 1100 °C, Ti(C,N) becomes the dominant phase, as it can stably incorporate small amounts of nitrogen; at 850 °C, titanium exhibits a greater tendency to combine with carbon to form TiC, likely attributable to diminished nitrogen diffusivity; at 600 °C, the low temperature inhibits atomic diffusion, thereby allowing for the stable retention of Ti(C,N) formed in earlier stages; and at 950 °C—a transitional range—both phases can coexist. Despite the extremely low nitrogen content in the steel (0.0031 wt%), titanium possesses a higher affinity for nitrogen than for carbon. At high temperatures (e.g., 1100 °C), titanium preferentially combines with the limited nitrogen to form Ti(C,N). When the temperature decreases to 850 °C, the remaining titanium mainly combines with carbon to form TiC, potentially because nitrogen has been mostly consumed or its solubility has diminished. At 950 °C, nitrogen distribution attains a dynamic equilibrium, leading to the coexistence of both phases. At high temperatures (e.g., 1100 °C), carbon and nitrogen atoms exhibit high diffusivity and can uniformly combine with titanium to form Ti(C,N). At medium and low temperatures (e.g., 850 °C, 600 °C), the atomic diffusion rate decreases, increasing the likelihood of compositional segregation and thus facilitating the stable existence of a single phase (either TiC or Ti(C,N)). At 950 °C, the moderate diffusion rate enables both phases to form and coexist. Both phases exhibit a predominantly rectangular morphology (with only Ti(C,N) at 600 °C displaying a hexagonal shape), indicating that the stability of the crystal structure serves an auxiliary function in their existence mode. Therefore, the precipitated phases in the sample were the Ti-rich precipitates Ti(C,N) or TiC. Figure 10 illustrates the morphologies and compositions of the precipitated phases at 600 °C, 850 °C, 950 °C, and 1100 °C, respectively. At 600 °C, the precipitates were identified as Ti(C,N) (Figure 10a,b), with either hexagonal or rectangular morphology. At 850 °C, the precipitated phase was TiC (Figure 10c), with a rectangular morphology. At 950 °C, the precipitates consisted of either Ti(C,N) or TiC (Figure 10d,e), both exhibiting rectangular shapes. At 1100 °C, the precipitated phase was Ti(C,N) (Figure 10f) with a rectangular shape. According to the findings of references [10,19,20], for steel grades with high aluminum content (1.33 wt%–1.57 wt% Al by mass fraction), the primary observed precipitates are AlN, Nb(C,N), TiN, VN, etc. Considering the compositional features of 38CrMoAl steel in this study (0.85 wt% Al, 0.0031 wt% N, 0.0127 wt% Ti by mass fraction), literature specifically investigating precipitates in 38CrMoAl steel is relatively limited; thus, high-aluminum steel grades were selected as reference materials. Multiple TEM characterizations were performed on carbon replica samples of this steel, and only Ti(C,N) or TiC precipitates were identified.

**Figure 11:**
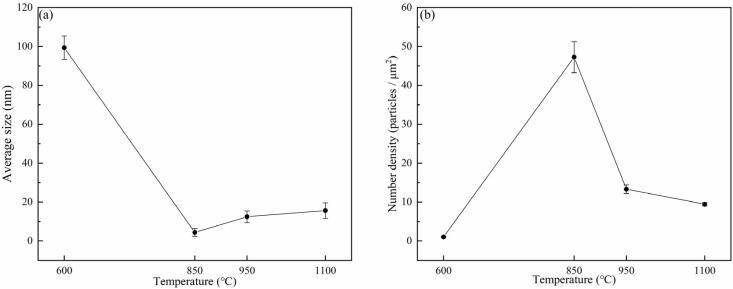
Average size and number density of precipitates in the tested steels: (**a**) mean size; (**b**) number density.

Figure 11 presents the variations in the average size and number density of Ti-rich precipitated phases at 600 °C, 850 °C, 950 °C, and 1100 °C, respectively. As depicted in Figure 11a, the average size of precipitates first decreased, then increased, and finally decreased again. Specifically, the maximum average size of precipitates occurred at 600 °C (~99.37 nm), while the minimum value (~4.39 nm) was observed at 850 °C. According to Figure 11b, the number density of precipitates exhibited an initial increase followed by a decrease, with a peak at 850 °C. The minimum number density was recorded at 600 °C (~1.03 particles/μm^2^), whereas the maximum value (~47.24 particles/μm^2^) was attained at 850 °C. The phenomenon where the size of precipitates decreases while their density increases is rooted in the dynamic equilibrium between the nucleation, growth, and dissolution of precipitates. At relatively high temperatures, atomic mobility is significantly enhanced, accelerating the diffusion of solute atoms and increasing the nucleation rate. The formation of a large number of new nuclei outweighs the losses from dissolution, thereby increasing the overall density. Elevated temperatures generate more heterogeneous nucleation sites, providing favorable conditions for precipitate nucleation. Within the transitional temperature range, the temperature is sufficient to activate rapid nucleation but not high enough to cause widespread dissolution of all precipitates. Here, the formation rate of new nuclei exceeds the dissolution rate of existing precipitates, leading to an increase in density. In the intermediate temperature range, the rate of new nucleation surpasses the ripening process. Although small precipitates may shrink, the rapid formation of new nuclei maintains or increases the overall density.

The increase in the precipitate quantity and the decrease in the precipitate size were detrimental to the hot ductility of steel [31]. The higher the number density of precipitates and the smaller their size, the more pronounced the deterioration in hot ductility [32,33]. Fine precipitation can increase the strength of the matrix, thus intensifying the stress at the boundaries, favoring grain boundary sliding. Moreover, when situated at the boundaries, it can make intergranular cracks interlink more easily [33].

During hot tension testing, the deformation mismatch between precipitates and the matrix resulted in strain concentration. This localized strain can induce significant ductility deformation and promote the nucleation of micro-voids in the precipitates [34]. These micro-voids can act as initiation sites for crack formation, further compromising the material’s hot ductility.

**Figure 12:**
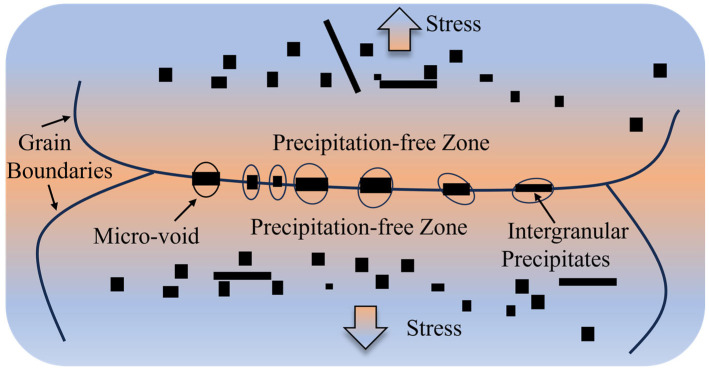
Mechanism of decrease in hot ductility by precipitates.

Figure 12 illustrates the mechanism of reduced hot ductility caused by the precipitates. Ti-rich precipitates were segregated at grain boundaries and formed a precipitate-free zone (PFZ) around the grain boundaries. Strain tended to concentrate near the grain boundaries under stress, primarily because the PFZ exhibited lower hardness [35]. Therefore, micro-voids formed on the grain boundary precipitates as the stress increased. After the voids coalesced, intergranular non-ductile fracture occurred.

**Figure 13:**
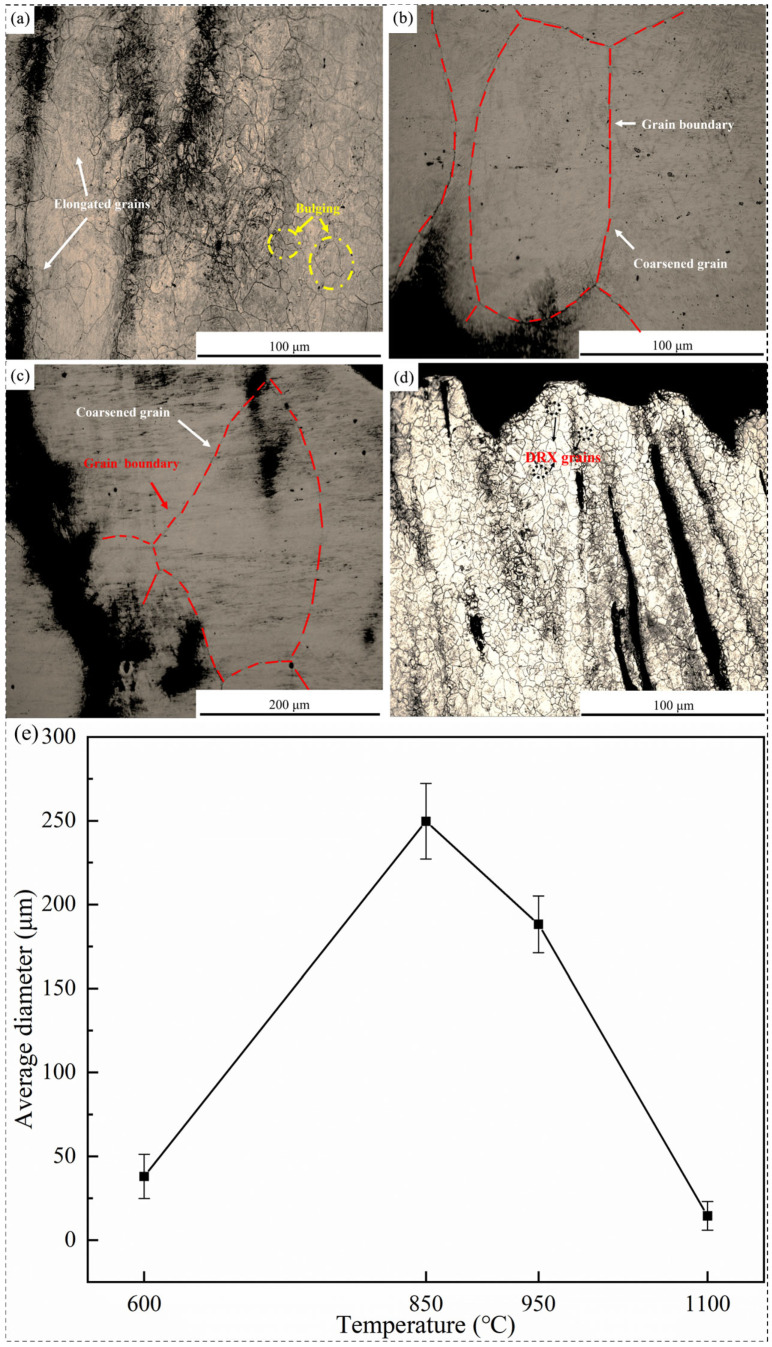
Grain morphology near the fracture surface and average grain size: (**a**) 600 °C; (**b**) 850 °C; (**c**) 950 °C; (**d**) 1100 °C. (**e**) Average grain size at 600 °C, 850 °C, 950 °C, and 1100 °C.

Grain sizes adjacent to the fracture surface were quantified using the Image-Pro Plus software, with the results presented in Figure 13. At 600 °C (Figure 13a), deformed grains exhibited elongation along the tensile axis, accompanied by the outward bulging of the grain boundaries, indicative of ductile deformation under low-temperature conditions. Coarsening was evident at 850 °C and 950 °C (Figure 13b,c), where larger equiaxed grains dominated, likely due to reduced deformation energy and prolonged thermal exposure promoting grain growth. At 1100 °C (Figure 13d), coarse deformed grains were replaced by fine dynamic recrystallization (DRX) grains, signaling active grain boundary migration and new grain nucleation under high-temperature conditions. Quantitative analysis (Figure 13e) revealed a non-monotonic grain size trend, increasing from 600 °C to 850 °C (peak average size: ~249.69 μm) before decreasing to ~14.47 μm at 1100 °C. This correlated with the ductility trend, where coarse grains at 850 °C exacerbated intergranular stress concentration (brittle behavior), while fine DRX grains at 1100 °C enhanced the plastic deformation capacity via improved grain boundary sliding and strain accommodation.

The relationship between precipitates and hot ductility is controlled by their size, number density, and interaction with grain boundaries. An increase in precipitate quantity and a decrease in precipitate size are detrimental to the hot ductility of steel [31]. This mechanism was evident across the four tested temperatures (600 °C, 850 °C, 950 °C, and 1100 °C), where the precipitate size and density exhibited inverse trends (Figure 11). When the temperature increased from 600 °C to 850 °C, the drastic reduction in precipitate size (from ~99.34 nm to ~4.40 nm) and simultaneous increase in number density (from ~1.03 to ~47.24 particles/μm^2^) enhanced precipitate pinning at grain boundaries, theoretically suppressing grain growth. Paradoxically, this regime experienced grain coarsening (from ~122.56 μm to ~249.69 μm), likely due to stress-driven boundary migration under constrained deformation. The fine, dense precipitates acted as stress concentrators, promoting intergranular cracking and reducing the RA from 57.5% to 26.1%, a classic brittle behavior associated with precipitate-induced grain boundary embrittlement. As the temperature rose from 850 °C to 1100 °C, precipitate coarsening and dissolution at higher temperatures weakened their pinning effect, enabling DRX to dominate. This led to significant grain refinement (from ~249.69 μm to ~14.47 μm), as DRX nucleated fine grains and eliminated coarse deformed structures. The reduced precipitate density (from ~47.24 to ~3.58 particles/μm^2^) and grain refinement enhanced deformation homogeneity and intergranular coordination, restoring RA values above 40%. These observations are consistent with the results reported in [36,37,38,39,40].

(ii) Microstructure

The microstructures of the specimens at four different temperatures are shown in Figure 8. At 600 °C, the specimen exhibited a Widmanstätten pattern with proeutectoid ferrite at grain boundaries, with an average thickness of approximately 2 μm. When the temperature increased to 850 °C, the microstructure consisted of martensite and proeutectoid ferrite at grain boundaries, with an average thickness of 15 μm. The strength of intergranular proeutectoid ferrite is generally lower than that of the austenite matrix [41]. During deformation, stress is concentrated in these proeutectoid phases, leading to the nucleation, growth, and aggregation of micro-voids at grain boundaries to form cracks, which reduces the hot ductility of steel. The thickness of ferrite at grain boundaries shows a negative correlation with RA values [42]. When the straightening stresses exceeded the critical strain, cracks initiated along the austenite grain boundaries. Consequently, the hot ductility at 600 °C was better than that at 850 °C. As the temperature rose from 850 °C to 950 °C and then to 1100 °C, the microstructure transitioned from the α + γ two-phase region to a single γ phase (as shown in Figure 9). The microstructure of the specimens at 950 °C and 1100 °C consisted of austenite, which transformed into martensite upon rapid cooling (as shown in Figure 8). From 850 °C to 950 °C, the proeutectoid ferrite at grain boundaries gradually dissolved into the γ phase, causing the microstructure to transition from the α + γ two-phase region to a single γ phase. As the temperature rose from 950 °C to 1100 °C and exceeded the critical temperature of the single-γ-phase region, the microstructure fully transformed into uniform austenite; its grain size was significantly influenced by temperature. When deformation occurred in the single-phase region above 950 °C, the absence of soft ferrite phase allowed stress to be uniformly distributed in the austenite matrix, reducing stress concentration at the grain boundaries. As a result, the hot ductility was significantly improved compared with the two-phase region (850 °C), with the RA value increasing from 26.1% to over 83.5%.

## 4. Effect of Heat Treatment on Precipitated Phase and Microstructure

Heat treatment critically regulates precipitated phase evolution and microstructural features in steel, with the holding time and cooling rate serving as key determinants [43]. A prolonged holding time tends to facilitate the coarsening of the precipitate and diminishes their number density, while rapid cooling suppresses proeutectoid phase formation and induces metastable structures. These modifications directly influence the intergranular stress distribution and DRX capacity, thereby governing hot ductility [44,45,46,47].

**Figure 14:**
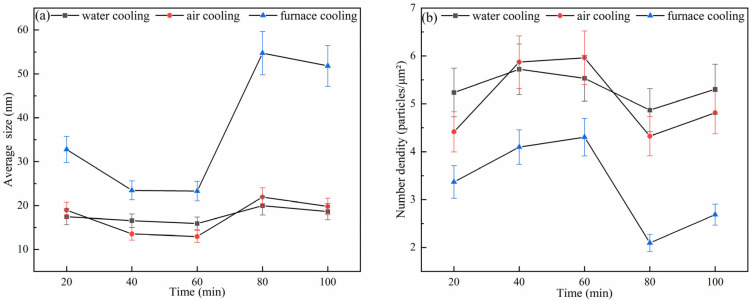
Average size and number density of precipitated phases under different holding times and cooling rates: (**a**) mean size; (**b**) number density.

Thus, this study investigated the effects of different holding times and cooling rates on the precipitated phases and microstructure of 38CrMoAl steel. The average size and number density of precipitated phases under varying holding times and cooling rates are shown in Figure 14.

Figure 14a reveals that under three cooling conditions (water cooling, air cooling, and furnace cooling), the average size of the precipitated phases exhibited a trend of decreasing–increasing–decreasing with increasing holding time: (1) at holding times of 20 min, 80 min, and 100 min, the order of the average size of the precipitated phases was furnace cooling > air cooling > water cooling; (2) at holding times of 40 min and 60 min, the order changed to furnace cooling > water cooling > air cooling. Figure 14b shows that the variation trend of the precipitate number density was inverse to that of the average size: (1) at holding times of 20 min, 80 min, and 100 min, the number density order was water cooling > air cooling > furnace cooling; (2) at holding times of 40 min and 60 min, the order became air cooling > water cooling > furnace cooling.

Water cooling inhibited precipitate coarsening and promoted the formation of fine precipitates with a high number density, which was particularly obvious at holding times of 20 min, 80 min, and 100 min. This was because the rapid cooling rate associated with water quenching reduced the average size of precipitates and enhanced their nucleation rate [48]. In contrast, furnace cooling prolonged atomic diffusion, leading to precipitate coarsening and a decrease in number density. At moderate holding times of 40 min and 60 min, the competition between dynamic recrystallization and precipitate formation under air cooling may generate a unique size distribution of precipitates. This is due to the interplay between the driving force for recrystallization and the pinning force of precipitates, which can lead to a more complex microstructural evolution [49].

A reduction in precipitate average size and an increase in number density are detrimental to hot ductility. Under the same holding time conditions, decreasing the cooling rate can promote the coarsening of the precipitate size and a reduction in the number density, which benefits the hot ductility of a specimen. In contrast, a higher cooling rate results in finer precipitates with a higher number density, which can impair hot ductility.

**Figure 15:**
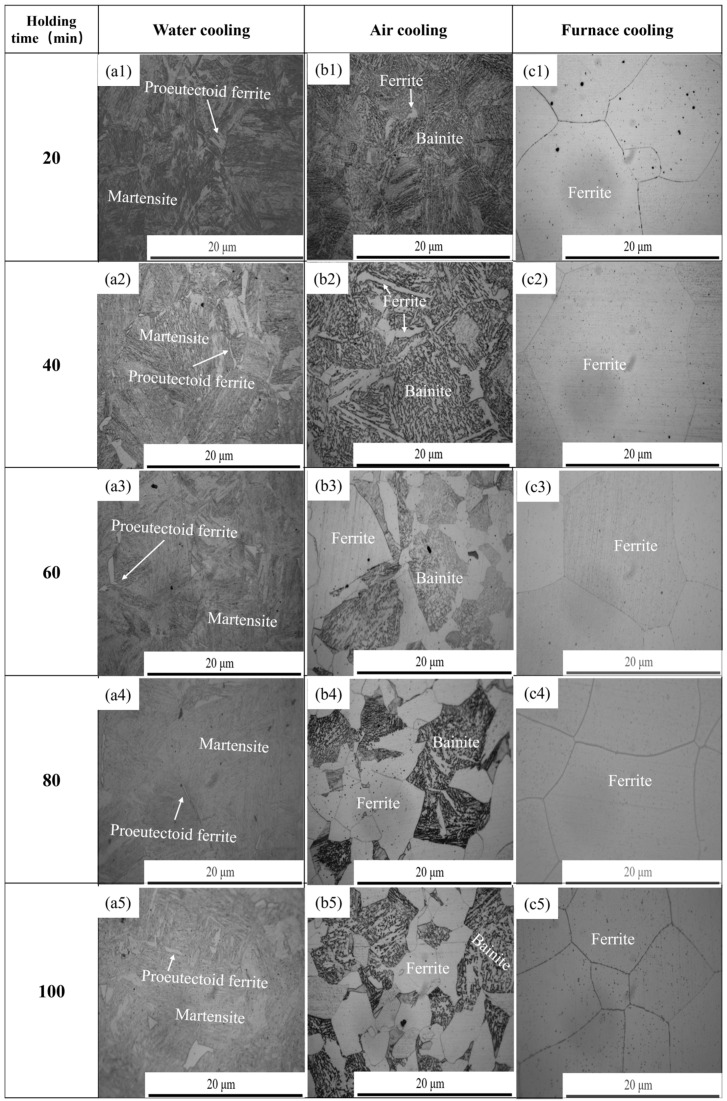
Microstructure of 38CrMoAl steel under different holding times and cooling rates: (**a1**–**a5**) holding time for 20 to 100 min under water cooling; (**b1**–**b5**) holding time for 20 to 100 min under air cooling; and (**c1**–**c5**) holding time for 20 to 100 min under furnace cooling.

Figure 15 illustrates the microstructural transformations in 38CrMoAl steel under different combinations of holding times and cooling rates. At a constant holding time, increasing the cooling rate (furnace cooling → air cooling → water cooling) drove the following phase transitions: ferrite → bainite + ferrite → martensite + proeutectoid ferrite. Factors such as microstructural inhomogeneity, minor temperature gradients caused by differences in heat transfer efficiency in small-sized samples, localized “hot spots” or slow-cooling zones that provide conditions for ferrite formation, and the ability of Al and Cr to stabilize ferrite and enhance its nucleation tendency collectively contribute to the formation of proeutectoid ferrite during water cooling.

Figure 15(a1–a5) shows that as the holding time increased from 20 min to 100 min, the thickness of proeutectoid ferrite precipitated along the grain boundaries exhibited a sequential trend of increasing, decreasing, and then increasing. As observed in Figure 15(b1–b5), the area fraction of ferrite increased with the prolongation of the holding time. Under furnace cooling conditions, the microstructure consisted of ferrite, as shown in Figure 15(c1–c5).

The hot ductility of steel is significantly influenced by its microstructure. When martensite and proeutectoid ferrite coexist in steel, martensite exhibits high hardness and poor ductility; a higher content of martensite will reduce the overall ductility of steel. If proeutectoid ferrite is distributed along grain boundaries in a coarse network morphology, it will disrupt the matrix continuity, weaken grain boundary bonding, and deteriorate hot ductility. When bainite and ferrite coexist, lower bainite can improve the hot ductility of steel due to its superior comprehensive mechanical properties. Ferrite itself has good ductility and toughness, and a higher proportion of ferrite helps enhance the deformation capacity of steel during hot ductility. For example, the steel grade studied in [50,51] forms a bainite + ferrite microstructure under specific cooling conditions, demonstrating excellent hot ductility characteristics. Ferrite has a significant impact on the hot ductility of steel, with its content, morphology, and distribution being crucial. Generally, uniform and fine ferrite microstructures can improve hot ductility.

Martensite—a hard and brittle phase—exhibits a lath or acicular morphology [52,53], contributing to localized stress concentration due to its low ductility. The formation of proeutectoid ferrite networks along grain boundaries disrupts matrix continuity, creating preferential paths for crack initiation during deformation. In contrast, bainite, characterized by needle-like or lath-shaped structures, forms a “soft–hard alternating” composite with ferrite. This heterogeneous structure balances strength and ductility, as the softer ferrite phase (predominantly in blocky or acicular forms with uniform distribution) facilitates plastic deformation, while bainite provides load-bearing capacity. Single-phase ferrite microstructures, with their uniform deformation behavior and low resistance to grain boundary sliding, significantly reduce crack susceptibility. For 38CrMoAl steel, decreasing the cooling rate under constant holding time conditions promotes the formation of coarse ferrite and bainite–ferrite mixtures rather than brittle martensite, thereby enhancing hot ductility via improved intergranular cohesion and deformation homogeneity.

## 5. Conclusions

To address the issue of casting slab cracking, this study investigated the high-temperature mechanical properties of 38CrMoAl steel to determine the brittle temperature region prone to cracking and the fracture type, analyze the causes of poor hot ductility in each temperature range, and research the heat treatment of 38CrMoAl steel. The main conclusions are as follows:

(1) The high-temperature tensile test of 38CrMoAl steel was carried out using the Gleeble-3800. Based on the criterion of RA = 40%, the steel did not exhibit the first and second brittle regions, and the temperature region of the third brittle was 645–1009 °C. Straightening or processing within the brittle temperature region of the casting slabs should be avoided as much as possible.
(2) Based on the fracture morphology, the specimens exhibited ductile fracture in the temperature ranges of 600 °C and 1050–1200 °C. In the temperature region of 650–750 °C, the specimens showed a combination of ductile fracture and intergranular brittle fracture. Within the temperature region of 800–1000 °C, the specimens underwent intergranular brittle fracture.
(3) The fracture mechanisms of 38CrMoAl steel are classified into three types: (I) in the α single-phase region, the thickness of intergranular proeutectoid ferrite increases with rising temperature, reducing hot ductility; (II) in the γ single-phase region, the average size of precipitates increases and the number density decreases with increasing temperature, improving hot ductility; and (III) in the α + γ two-phase region, the precipitation of proeutectoid ferrite facilitates crack propagation, and the dense distribution of precipitates at grain boundaries causes stress concentration, deteriorating hot ductility.
(4) The analysis of heat treatment for 38CrMoAl steel showed that with the same holding time, the microstructure of the specimen transformed as the cooling rate increased as follows: ferrite → bainite + ferrite → martensite + proeutectoid ferrite. Under three cooling conditions (water cooling, air cooling, and furnace cooling), the average size of precipitates exhibited a trend of decreasing first, then increasing, and decreasing again with the increase in holding time. (I) At holding times of 20 min, 80 min, and 100 min, the order of the average sizes of precipitates was furnace cooling > air cooling > water cooling. (II) At holding times of 40 min and 60 min, the order changed to furnace cooling > water cooling > air cooling. The variation trend of the precipitate number density was opposite to that of the average size.

## Figures and Tables

**Table 1:** The main chemical composition of 38CrMoAl steel (mass fraction, wt%).

C	Si	Mn	P	S	O	N	Ti	Cr	Mo	Al	Fe
0.39	0.31	0.42	0.0130	0.0010	0.0005	0.0031	0.0127	1.53	0.10	0.85	Bal.

**Table 2:** Phase characteristics of the experimental steel.

Precipitation	Starting Precipitation Temperature (°C)	Full Precipitation Temperature (°C)	Maximum Precipitation Amount (Volume Fraction)
α	870	-	0.932
γ	1474	740	1
Liquid	-	1444	1

## Data Availability

The data presented in this study will be made available upon request from the corresponding author. The data are not publicly available due to them involving trade secrets.

## Abbreviations

The following abbreviations are used in this manuscript:

RA	Reductions in area
PFZ	Precipitate-free Zone
DRX	Dynamic recrystallization
